# Analysis of high-intensity interval training on bone mineral density in an experimental model of type 2 diabetes

**DOI:** 10.1590/acb370207

**Published:** 2022-05-02

**Authors:** Letícia Alves Paiva, Iandara Schettert Silva, Silvio Assis de Oliveira, Albert Schiaveto de Souza, Claudio Osório Brito Jacques

**Affiliations:** 1 Master. Universidade Federal do Mato Grosso do Sul – Faculty of Medicine – Postgraduate Program in Health and Development – Campo Grande (MS), Brazil.; 2 PhD. Universidade Federal do Mato Grosso do Sul – Faculty of Medicine – Postgraduate Program in Health and Development – Campo Grande (MS), Brazil.; 3 PhD. Universidade Federal do Mato Grosso do Sul – Integrated Institute of Health – Postgraduate Program in Health and Development – Campo Grande (MS), Brazil.; 4 PhD. Universidade Federal do Mato Grosso do Sul – Biosciences Institute – Postgraduate Program in Health and Development – Campo Grande (MS), Brazil.; 5 Nutritionist. Universidade Federal do Mato Grosso do Sul – Faculty of Pharmaceutical Sciences – Research and Production Laboratory– Campo Grande (MS), Brazil.

**Keywords:** Diabetes Mellitus, Experimental, Osteoporosis, Bone Density, Rats

## Abstract

**Purpose:**

To analyze the effect of high-intensity interval training (HIIT) on bone mineral density (BMD) in a model of type 2 diabetes mellitus.

**Methods:**

Thirty-two male, adult, 12-week-old rats (*Rattus norvegicus*), of the Wistar lineage, were used. The animals induced to the experimental model received a high fat diet for 10 days and, after that period, intraperitoneal injection of streptozotocin (40 mg·kg^–1^), dissolved in 20 mmol·L^–1^ sodium citrate solution (pH = 4.5). The experimental group of diabetes was formed by the animals that, 48 h after the injection of streptozotocin, had fasting blood glucose > 250 mg·dL^–1^). The animals were randomly divided into four groups with eight animals each: HIIT experimental diabetes; HIIT control; sedentary experimental diabetes and sedentary control. The animals in the HIIT group performed an aerobic exercise protocol on a treadmill inclined at an angle of 15° to the horizontal, with interspersed intensity. Five weekly sessions, lasting 49 min each, were held for 6 weeks. The analysis of cortical bone density (CBD) and BMD were performed by X-ray images using the In-Vivo Xtreme II/Bruker system.

**Results:**

For CBD and BMD, when comparing diabetes and control groups, a significant difference was seen between groups in relation to HIIT (p = 0.007). Animals submitted and not submitted to HIIT in the same group showed a significant difference between groups in relation to diabetes (p < 0.001).

**Conclusions:**

The HIIT experimental diabetes group had increased CBD and BMD in comparison with the sedentary experimental diabetes group.

## Introduction

The population of people with diabetes in the world are estimated to be 422 million, the majority living in low and middle income countries^1^. Type 2 diabetes stands for about 90–95% of diabetics^2^.

Several studies address the complications of diabetes mainly with regard to metabolic complications; however, osteoporosis has compromising factors in its diagnosis and prevention, for example, the decreased bone mineral density (BMD) in the case diabetes type 1; and normal and/or increased, in diabetes type 2^3^–^5^. Bone strength is defined by BMD and bone quality, and its impairment is characteristic of osteoporosis skeletal disorder^6^. Studies show that type 2 diabetes can negatively affect the cortical structure of the bone and reduce bone strength by accumulating advanced glycation end (AGE) products in the bone^6^^,^ 7.

Exercises can be effective in stimulating bone osteogenesis in osteoporosis^8^. Regular physical exercise can be efficient in maintaining or increasing BMD; however, studies show that not all exercise modalities are equally osteogenic^9^^,^ 10. Studies that employed running and resistance training in type 2 diabetic rats have obtained positive results related to BMD^6^^,^ 8.

High-intensity interval training (HIIT) consists of alternating short periods of exercise and periods of high intensity^11^. In the literature, there are studies that demonstrate satisfactory results of this exercise modality in several pathophysiological aspects, such as improved cardiac rehabilitation in cardiac transplant patients^12^, reduced blood pressure at rest in hypertensive adults^13^, and reduced fasting glycemia and glycated hemoglobin in patients with type 2 diabetes^14^. There is evidence that HIIT improves BMD in postmenopausal women, when there are hormonal changes that contribute to functional decline and to alterations in body composition and bone metabolism^9^. However, the pathology of diabetic osteoporosis is different from postmenopausal osteoporosis^15^. Thus, there are few studies in the literature that correlate HIIT in diabetic osteoporosis with its results regarding BMD.

## Methods

All experimental procedures were performed according to the *Guide for the Care and Use of Laboratory Animals*, published by U. S. National Institutes of Health, and according to the *Guidelines of the National Council for the Control of Animal Experimentation* (CONCEA). The work was approved by the Ethics Committee on the Use of Animals / CEUA UFMS No. 1,047/2019.

Thirty-two male adult (12-week-old) rats (*Rattus norvegicus*) of the Wistar lineage were used. They were kept at the vivarium (UT / Center for Biological and Health Sciences, Federal University of Mato Grosso do Sul, Campo Grande, Brazil) under controlled conditions of light (12 h light / dark cycle) and temperature (22 ± 2 °C; 40 to 60% relative humidity), with food and water ad libitum.

**Figure 1:**
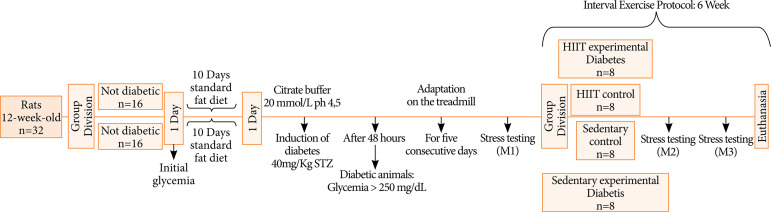
Study design.

The animals induced to the experimental model of diabetes received a high fat diet (50% fat, 13% protein and 37% carbohydrate) for 10 days. After 10 days, the animals received an intraperitoneal injection of streptozotocin (40 mg·kg^–1^; Sigma Chemical, St. Louis, MO, USA) dissolved in a 20 mmol·L^–1^ sodium citrate solution (pH = 4.5). The streptozotocin dosage performed in this study for the experimental model of type 2 diabetes used, simulates metabolic characteristics of DM2 in humans^16^. Blood glucose was measured 48 h after the injection, and fasting glycemia > 250 mg·dL^–1^ was considered hyperglycemia. Blood glucose was determined by using a colorimetric enzyme assay (ACCU-CHEK Performa; Roche Diagnostics Brazil Ltd., São Paulo, Brazil). The animals of the nondiabetic control group received the standard ration for 10 days. The animals were randomly divided into four groups, each containing eight animals: HIIT experimental diabetes group; HIIT control group; sedentary experimental diabetes group and sedentary control group. The animals in the HIIT group performed a high-intensity interval exercise protocol^17^. After 48 h of the last training, the animals were anesthetized with intraperitoneal injection of ketamine (10 mg·kg^–1^) and xylazine (2 mg·kg^–1^). Euthanasia was performed with sodium pentobarbital (100 mg·kg^–1^). The trial period totaled 6 weeks, as shown in Fig. 1.

### Stress test and interval exercise protocol

One week before the application of the stress testing and onset of the exercise protocol, there was a period of adaptation on the treadmill, for five consecutive days, with a constant speed of 10 m·min^–1^ and increasing duration of 10, 15, 20, 25 and 30 min, respectively.

To assess functional capability, a maximum effort test was performed using an incremental protocol for rats^17^ in order to determine the maximum speed achieved. The stress test was carried out in three phases: at the beginning (M1), at the end of week 4 (M2) and at the end of the experimental period that is, ending of week 6 (M3).

The stress test started with a 5-min warm-up at a speed of 5 m·min^–1^. After 3 min of passive recovery, the animal was submitted to the progressive effort test, with an initial speed of 6 m·min^–1^, with running stages under constant load and increments of 3 m·min^–1^ between the subsequent stages, which was performed at every 3 min. The protocol ended when the animal reached exhaustion, which was defined when it refused to run, even under physical stimulation with a light touch on the animal’s back or when the coordination between steps was difficult.

Subsequently, the protocol of interval aerobic physical exercise began, consisting of the treadmill running model with interspersed intensities. Five weekly sessions of physical exercise were carried out, lasting 49 min each. The cycles comprised of 3 min at 60% of the maximum speed achieved in the exercise test, followed by 4 min of running at 85% of the maximum speed; the cycles were repeated seven times. The protocol was performed with the treadmill inclined at an angle of 15° to the horizontal^17^. A preheating to each session occurred at an intensity of 40% of the maximum speed reached for 10 min.

### Evaluation of bone mineral density

After euthanasia, the right legs were removed and stored at a temperature of –80 °C. The X-ray energy analyzes were obtained by using the In-Vivo Xtreme/Bruker II system (Biosoin Bruker, Ettlingen, Germany), with the Molecular Imaging Software program. The computerized microtomography device is located at the Laboratory of Studies in Experimental Models of Diseases (LAEME) of the UFMS. For analysis, the forelegs were positioned in a prone position with an elbow angle of 90° inside the In-Vivo Xtreme/Bruker device. To measure cortical bone density (CBD) and BMD, the humerus bone was used and divided into six regions of interest (ROI), positioned in the proximal and distal epiphyses; and longitudinal diaphysis in each bone. Values in g·cm^–2^ (CBD) and g·cm^–3^ (BMD) were expressed as the mean and standard error of the six points of each humerus bone^18^.

### Statistical analysis

The evaluation of the effects of diabetes and HIIT, as well as the interaction between these factors in relation to BMD, was performed using the two-way ANOVA test, followed by the Tukey’s post-test. The comparison between animals not submitted to HIIT and those subjected to this treatment, in animals of the diabetes group, was drawn using the Student’s t-test. The other results evaluated in this study were presented in the form of graphs. The statistical analysis was performed using the statistical program SigmaPlot, version 12.5, considering a significance level of 5%^19^.

## Results

**Figure 2:**
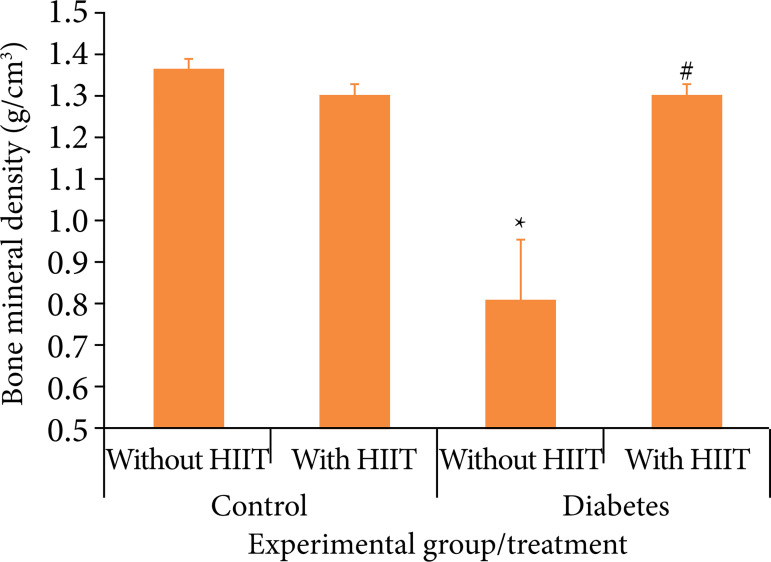
Bone mineral density in each experimental group and applied treatment. Each column represents the mean and the bar the standard error of the mean.

**Figure 3:**
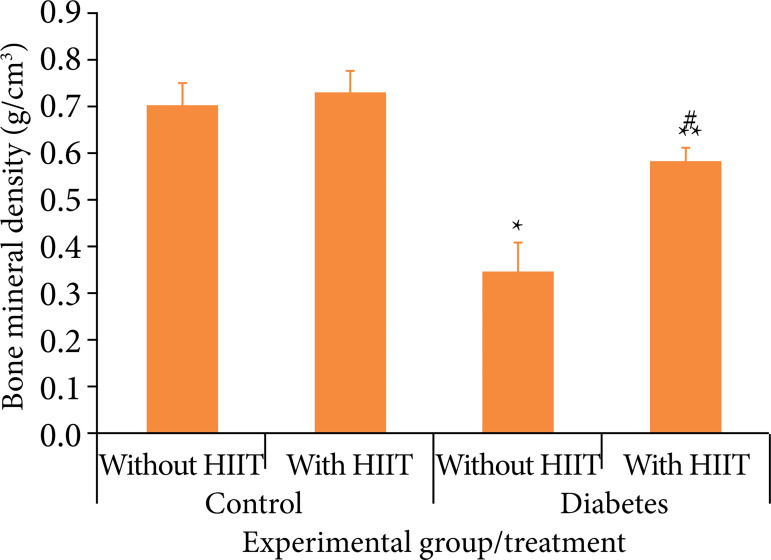
Cortical bone density in each experimental group and applied treatment. Each column represents the mean and the bar the standard error of the mean.

The results of the analysis of diabetes and HIIT, in relation to CBD and BMD are presented in Figs. 2 and 3 and Table 1.

*Significant difference compared to the control group, without HIIT (Tukey’s post-test,p < 0.05); #Significant difference compared to diabetic animals without HIIT (Tukey’s post-test, p < 0.05).

*Significant difference compared to the control group, without HIIT (Tukey’s post-test, p < 0.05);**Significant difference in relation to the control group, with HIIT (Tukey’s post-test, p < 0.05);#Significant difference in relation to diabetic animals without HIIT (Tukey’s post-test, p < 0.05).

**Table 1:** Results of the assessment of the effect of diabetes and HIIT, as well as the interaction between these factors, in relation to the variables evaluated in this study.

**Variable**	**Control**			**Diabetes**		**Value p**
	**Without HIIT**	**With HIIT**		**Without HIIT**	**With HIIT**	
BMD (g·cm^–3^)	1.36 ± 0.03Aa	1.30 ± 0.03Aa		0.81 ± 0.14Bb	1.30 ± 0.03Aa	DM < 0.001; HIIT = 0.007; Inter. < 0.001
CBD (g·cm^–2^)	0.70 ± 0.04Aa	0.74 ± 0.04Aa		0.34 ± 0.6Bb	0.58 ± 0.3Aa	DM < 0.001; HIIT = 0.005; Inter. < 0.001
Weight (1st week) (g)	212.58 ± 3.90	215.50 ± 3.74		206.00 ± 4.88	208.67 ± 3.52	DM = 0.104; HIIT = 0.494; Inter. = 0.975
Weight (5th week) (g)	322.42 ± 5.82A	309.42 ± 6.64A		265.17 ± 8.27B	264.00 ± 8.25B	DM < 0.001; HIIT = 0.339; Inter. = 0.423
Weight (8th week) (g)	374.83 ± 8.10A	355.75 ± 8.45A		300.25 ± 14.31B	308.25 ± 9.90B	DM < 0.001; HIIT = 0.600; Inter. = 0.203

DM = effect of diabetes; HIIT = effect of HIIT; Inter. = Interaction between diabetes and HIIT factors.

The results are presented as mean ± standard error of the mean. P values in the two-way ANOVA test mean as follows: DM = effect of diabetes; HIIT = effect of HIIT; Inter. = Interaction between diabetes and HIIT factors. Different capital letters on the line indicate a significant difference between the control group and the diabetes group, in the same treatment in relation to HIIT (Tukey’s post-test, p < 0.05). Different lowercase letters on the line indicate a significant difference between animals not submitted and those submitted to HIIT, in the same group in relation to diabetes (Tukey’s post-test, p < 0.05).

In CBD, when comparing the diabetes and control groups, a significant difference was observed between the groups in relation to HIIT (p = 0.007). When comparing animals submitted and not submitted to HIIT in the same group, a significant difference was observed in relation to diabetes (p < 0.001). The interaction between diabetes and HIIT factors showed a significant difference (p < 0.029).

In BMD, when comparing the diabetes and control groups, a significant difference was seen between the groups in relation to HIIT (p = 0.007). When animals submitted and not submitted to HIIT are compared in the same group, a significant difference was observed in relation to diabetes (p < 0.001). The interaction between diabetes and HIIT factors showed a significant difference (p < 0.001).

**Figure 4:**
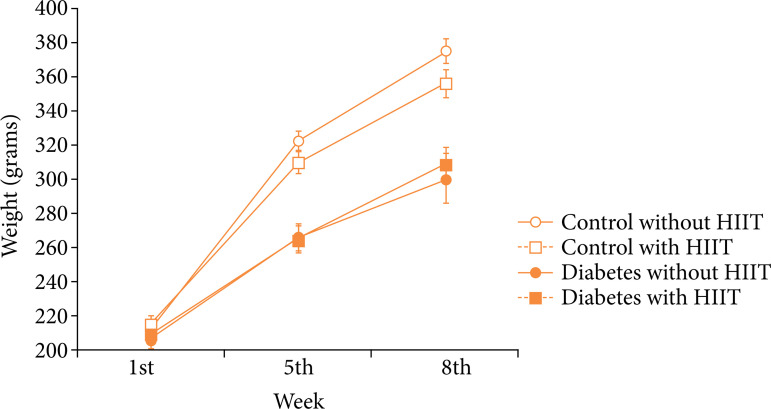
The weight of the animals in each treatment, in the different weeks evaluated in the study. Each symbol represents the mean, and the bar stands for the standard error of the mean.

The representation of the animals’ weight in each treatment in the different weeks of study lies in Table 1 and Fig. 4. A significant difference can be seen in weight in relation to the diabetes and control groups (p < 0.001) throughout the study.

## Discussion

This study demonstrated a decrease in CBD in sedentary diabetic animals and an increase in mineral bone density in diabetic animals undergoing HIIT. Studies of patients with type 2 diabetes report increased risk of bone fractures, although they present normal and/or high BMD^3^^,^ 5 ^,^ 7 ^,^ 20. Patients with type 2 diabetes show a trabecular bone score lower than that of nondiabetic individuals, and this lower score may be associated with an increased risk of fracture in diabetic patients^5^. The increased BMD in type 2 diabetic individuals was related to the high body mass index and elevated levels of glycated hemoglobin (HbA1c)^3^. In the present study, the diabetic animals showed lower body weight from the 5th week of the experiment when compared to the weight of the animals in the control group (p < 0.001), as shown in Fig. 4. Type 2 diabetic patients have greater cortical porosity when compared to nondiabetics. Serum concentrations of carboxylated and noncarboxylated osteocalcin (bone formation marker) and noncarboxylated C-terminal telopeptide (bone resorption marker) were found in lower concentrations in type 2 diabetics when compared to nondiabetics. Hyperglycemia and increased oxidative stress in patients with diabetes mellitus are responsible for increasing the levels of AGEs in the bone, responsible for reducing bone formation by inhibiting the synthesis of type 1 collagen and osteocalcin. The activation of the AGE receptor (RAGE) expressed in cells derived from bone cells can increase the production of inflammatory cytokines and reactive oxygen species that would result in chronic inflammation and bone resorption^3^^,^ 20.

Type 2 diabetic patients present a reduced bone turnover, and the accumulation of AGEs in the bone contributes to reduced bone resistance^7^. In the study of type 2 diabetes, in relation to BMD and bone turnover in men and women, the authors also found an increased risk of fracture with the weakening of bone structure in type 2 diabetes mellitus, despite normal and/or increased BMD. The study corroborates the need for further studies to support the pathophysiological mechanisms involved in this paradox. The low bone regeneration in type 2 diabetes would be associated with the mechanisms of low levels of the C-terminal telopeptide marker of bone resorption and the osteocalcin bone formation marker^20^.

The study on bone microstructure in diabetic mice found a reduction in spongy bone tissue and cortical bone thickness when compared to animals in the control group^21^.

Individuals with diabetes mellitus 2 presented higher trabecular bone density, but lower CBD, resulting in less bone resistance compared with nondiabetic individuals^5^.

Diabetes decreases bone strength, possibly because of changes in the cortical bone. The results of that study showed that cortical density was significantly lower in the diabetes group than in the control group^22^. The cortical density datum of that study corroborates that found in this study, where sedentary diabetic animals also had lower CBD compared with animals in the sedentary control group.

A six-week resistance training protocol resulted in significantly higher tibial BMD in a model of type 2 diabetic rodent when compared with control rats. There was also a significant increase in bone area and cortical thickness in animals submitted to resistance training in comparison with those in the control group. The study concluded that resistance training for six weeks effectively increased BMD and improved bone quality in type 2 diabetic rats^6^. In this study, an increase in CBD in diabetic animals submitted to HIIT for six weeks when compared with diabetic animals in the sedentary group was also observed.

In a study of three-month-old female rats with diabetes mellitus, the authors concluded that in the untreated diabetic group, there was a dramatic reduction in BMD and in the morphometric parameters of the femur of these animals^23^. In the present study, a reduction in BMD in the foreleg of diabetic animals that did not have HIIT was also found. The reduction of BMD in diabetic animals corroborates the studies that applied streptozotocin to induce diabetes mellitus^24^^,^ 25.

In the study on BMD and bone microarchitecture in diabetic mice, the authors found a decreased trabecular bone mass and BMD in diabetic animals when compared with animals in the control group^26^. The experimental diabetes model used in that study is similar to the diabetic model used in this study in comparison with the models of the aforementioned studies; however, trabecular bone mass was not evaluated.

Diabetic osteoporosis is characterized by a metabolic disorder of calcium and phosphorus, a decrease in BMD and changes in bone microstructure owing to relative or absolute insulin deficiency. Hyperglycemia favors the inhibition of the level of type I collagen and the mineralization of osteoblasts in studies with mice^25^. The literature presents divergences in relation to BMD in diabetic osteoporosis; however, most studies demonstrate that, in patients with type 2 diabetes mellitus, BMD is often normal and/or high. Changes in cortical and/or trabecular bone density can occur in type 2 diabetic individuals, which contributes to greater fragility of the bone structure of these individuals. The longer the duration of type 2 diabetes, the greater the fracture risk. Hyperglycemia, oxidative stress and the formation of AGE products have a direct effect on bone metabolism^27^. High glycemic levels and the use of some antidiabetic medications are some factors that have adverse effects on the skeleton, contributing to greater risks of fractures and/or bone diseases associated with type 2 diabetes mellitus^7^.

In this study, data on CBD and BMD in animals with and without type 2 diabetes mellitus were obtained. A significant increase in CBD was found in diabetic animals undergoing HIIT when compared with sedentary diabetic ones, and a significant increase of BMD in the HIIT diabetic group in relation to sedentary diabetic animals. The data presented in this study demonstrate that HIIT probably triggered a stimulus to bone osteogenesis in diabetic animals.

This study has some limitations. Trabecular bone mass, bone markers, AGE products and oxidative stress, analyzes directly related to bone metabolism, were not evaluated. Future studies are necessary for a better understanding of the repercussions of HIIT on the bone structure (bone quality and strength) of these patients.

## Conclusion

The high-intensity six-week interval training increased CBD and BMD in an experimental model of type 2 diabetes mellitus. This result indicates HIIT as a prevention and/or treatment strategy for diabetic osteoporosis and other possible comorbidities of diabetes already mentioned in the literature.
